# On the self-damping nature of densification in photonic sintering of nanoparticles

**DOI:** 10.1038/srep14845

**Published:** 2015-10-07

**Authors:** William MacNeill, Chang-Ho Choi, Chih-Hung Chang, Rajiv Malhotra

**Affiliations:** 1 Department of Mechanical Engineering, Oregon State University, Corvallis, Oregon, USA; 2 Department of Chemical Engineering, Oregon State University, Corvallis, Oregon, USA

## Abstract

Sintering of nanoparticle inks over large area-substrates is a key enabler for scalable fabrication of patterned and continuous films, with multiple emerging applications. The high speed and ambient condition operation of photonic sintering has elicited significant interest for this purpose. In this work, we experimentally characterize the temperature evolution and densification in photonic sintering of silver nanoparticle inks, as a function of nanoparticle size. It is shown that smaller nanoparticles result in faster densification, with lower temperatures during sintering, as compared to larger nanoparticles. Further, high densification can be achieved even without nanoparticle melting. Electromagnetic Finite Element Analysis of photonic heating is coupled to an analytical sintering model, to examine the role of interparticle neck growth in photonic sintering. It is shown that photonic sintering is an inherently self-damping process, i.e., the progress of densification reduces the magnitude of subsequent photonic heating even before full density is reached. By accounting for this phenomenon, the developed coupled model better captures the experimentally observed sintering temperature and densification as compared to conventional photonic sintering models. Further, this model is used to uncover the reason behind the experimentally observed increase in densification with increasing weight ratio of smaller to larger nanoparticles.

In photonic sintering broad-spectrum, pulsed or continuous light from a xenon lamp is incident onto nanoparticles deposited on a substrate. The optical energy is converted into heat by the nanoparticles, resulting in rapid evaporation of the solvent and in densification of the nanoparticles. The key advantage of photonic sintering over conventional nanoparticle sintering processes is faster densification of nanoparticle inks over large area substrates under ambient conditions^1^,^2^,^3^. Photonic sintering of a variety of nanoparticle materials has been demonstrated for applications in RFID tags, solar cells^1^,^4^, displays^5^,^6^,^7^ and flexible electronics^2^. Past work in pulsed photonic sintering has examined the effect of various optical parameters on densification. Increasing the number of exposures of the nanoparticles to the xenon lamp light reduces the electrical resistance of the sintered nanoparticles, indicating an increase in density of the sintered nanoparticles^8^,^9^,^10^,^11^. However, beyond a certain number of exposures there is little further increase in the density. Increasing the light energy per exposure and the duration of the exposure results in faster densification. Also, for a given optical power and exposure time, smaller nanoparticles result in higher conductivity and density of the sintered material^6^,^12^. A common hypothesis forwarded for the effect of nanoparticle size is that nanoparticle melting during photonic sintering is necessary for achieving high density, and smaller nanoparticles result in higher density due to the size dependent melting point of nanoparticles. Further, the properties of the sintered nanoparticles depend significantly on the sintered density^13^,^14^,^15^, and the substrate temperature and the sintered density should depend on the temperature rise in the nanoparticles. Park *et al.*^16^ used a thermocouple to measure the temperature rise in Cu nanoparticles dispersed with PVP during pulsed photonic sintering. A mesoscale heat transfer based model was developed that predicted the experimentally observed temperature evolution of the nanoparticles fairly well. One issue with this model is the assumption that all the incident optical energy is absorbed by the deposited nanoparticles. This precludes the well known wavelength dependent absorption of nanoparticles and the variation in optical power output of xenon lamp light with wavelength. West *et al.*^17^ combined effective medium theory, with a thermal transfer model and known optical power, to model the interaction between heat generation at the surface and heat conduction into the bulk of the deposited nanoparticles. Kim *et al.*^18^ measured light absorption by the as-deposited nanoparticles and combined it with known nanoparticle mass and incident optical power to predict nanoparticle melting. All of the above models make two key assumptions about the physics of photonic sintering. First, there is no neck growth between the nanoparticles, i.e., there is no change in the nanoscale morphology of the deposited nanoparticle ensemble, before melting occurs. Second, photonic heating is assumed to be constant throughout the process, i.e., even if there is neck growth it does not affect the magnitude of photonic heating. Past work in conventional nanoparticle sintering^19^,^20^,^21^ has demonstrated solid-state neck growth between nanoparticles that are initially in contact with each other and are subsequently subjected to a temperature rise. This neck growth changes the overall geometric shape of the nanoparticle ensemble. Further, past work in thermoplasmonics^22^,^23^,^24^,^25^,^26^,^27^ has shown that the magnitude of photonic heating depends on the shape of nanoparticles. These observations suggest that neglecting interparticle neck growth, and its effect on photonic heating during the process, may be an oversimplification of the physics of photonic sintering.

This work aims to understand the relationship between photonic heating, the mechanism of densification and the temperature rise of the nanoparticles during photonic sintering. A continuous xenon light source is used for photonic sintering of silver (Ag) nanoparticles. The temperature rise and densification are experimentally characterized in terms of the nanoparticle size. Further, Ag nanoparticles of different sizes are mixed together, and the effect of the weight ratio of smaller to larger nanoparticles on sintering temperature and densification is characterized. A nanoscale computational model of photonic sintering, that links electromagnetic Finite Element Analysis (FEA) with analytical models of interparticle neck growth, is developed to uncover the possibility and the nature of the coupling between interparticle neck growth and photonic heating. The predicted temperature evolution of the deposited nanoparticles during photonic sintering is compared to experimental observations. This model is further used to understand the effect of the aforementioned coupling, of modeling assumptions in literature, and of nanoparticle size, on temperature evolution and densification in photonic sintering.

## Results

### Experiments

**Figure 1:**
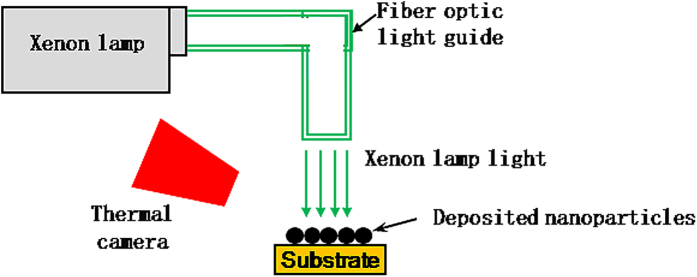
Schematic of the experimental setup for photonic sintering used in this work.

A custom-made experimental setup (schematically shown in Fig. 1) was used for photonic sintering of Ag nanoparticle inks with three different nominal diameters, i.e., 10 nm, 20 nm and 40 nm. Mixed nanoparticle inks with mixtures of (1) 10 nm and 20 nm and (2) 10 nm and 40 nm diameter nanoparticles in ratios by weight of 1:4, 2:3, 3:2 and 4:1 were also photonically sintered. The total solids loading content of all the inks was 50% by weight. No dispersant was used. A single droplet of nanoparticle ink was deposited onto a stainless steel substrate using a micropipette. A continuous xenon lamp was used as the light source for photonic sintering of the deposited droplet, with a fiber optic light guide used to direct the light to the deposited ink. The distance between the outlet of the light guide and the substrate, the commanded optical power and the lamp on-time, were fixed at the same value for every sintering experiment. The temperature of the deposited nanoparticles during sintering was monitored using a thermal camera. Further details of the experimental setup and procedures are provided in the methods section.

**Figure 2:**
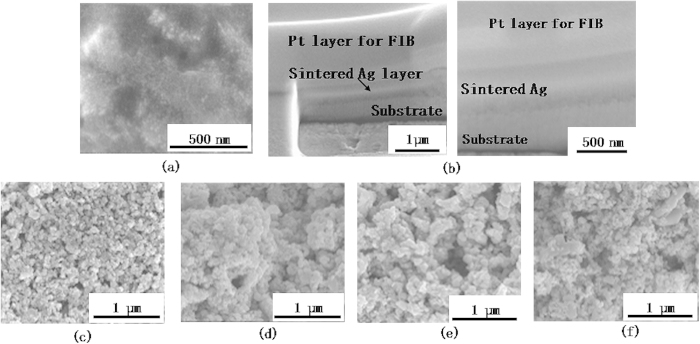
SEM images of (a) unsintered 10 nm ink (b) cross section of sintered 10 nm ink (c) unsintered 20 nm ink (d) sintered 20 nm ink (e) unsintered 40 nm ink (f) sintered 40 nm ink.

**Figure 3:**
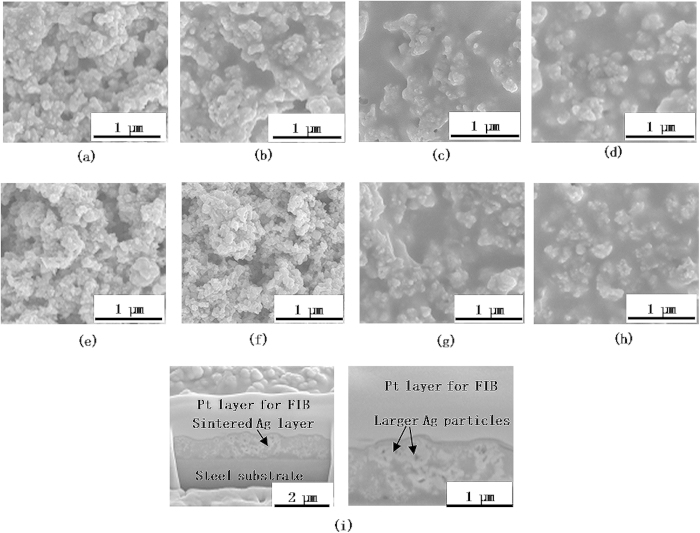
SEM images of sintered mixed 10 and 20 nm ink with weight ratio (a) 1:4 (b) 2:3 (c) 3:2 (d) 4:1; SEM images of sintered mixed 10 and 40 nm ink with weight ratio (e) 1:4 (f) 2:3 (g) 3:2 (h) 4:1; (i) cross sectional SEM image of sintered mixed 10 nm and 20 nm ink mixed in weight ratio 4:1.

**Figure 4:**
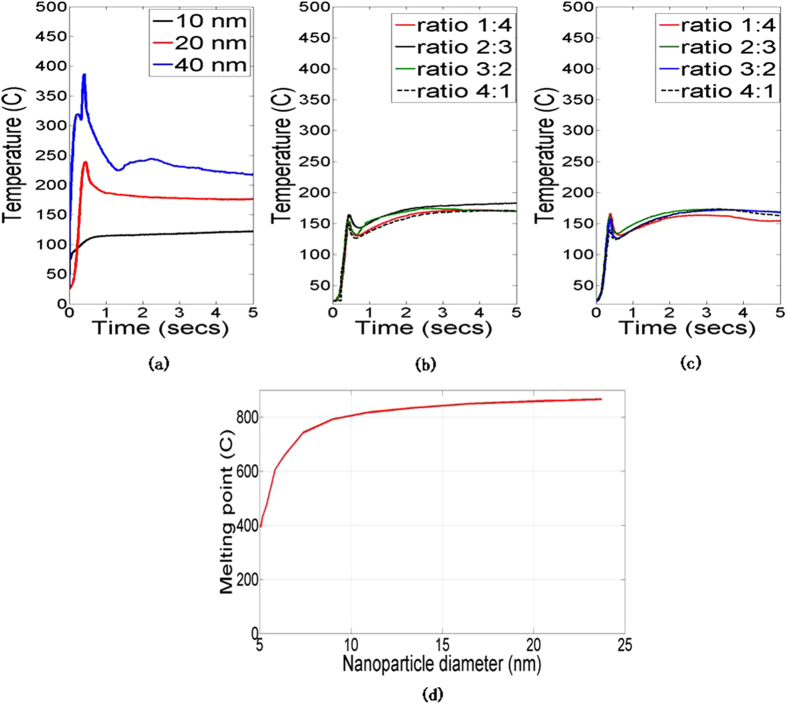
Evolution of maximum temperature of deposited nanoparticles during photonic sintering of (a) unmixed nanoparticles (b) 10 nm and 20 nm mixed nanoparticles (c) 10 nm and 40 nm mixed nanoparticles; and (d) melting point of silver as a function of nanoparticle diameter^28^.

Figure 2 shows SEM images of the unsintered and sintered nanoparticles, for the unmixed nanoparticle inks. Note that the 10 nm nanoparticles densify to a greater degree as compared to the larger nanoparticles. Larger scale SEM images of the sintered nanoparticles (in Supplementary Fig. S1 online) also reflect this phenomenon. Since the applied optical power and sintering time are the same for all the inks, it can be inferred that the 10 nm nanoparticles undergo faster densification than the larger nanoparticles. SEM images of the sintered nanoparticles for mixed 10 nm and 20 nm nanoparticles (Fig. 3) show that an increase in weight ratio of smaller to larger nanoparticles also results in faster densification. Larger scale SEM images are shown in Supplementary Figs S2 and S3 online. As the mixing ratio increases the morphology of the sintered material changes from one similar to that of the unmixed larger nanoparticle inks, to a morphology in which the larger nanoparticles are enclosed in a matrix of highly densified material. Figure 4 shows the evolution of the maximum temperature of the nanoparticles during photonic sintering. The peak temperature is higher for larger nanoparticles (Fig. 4a) and mixing nanoparticles of different sizes (Fig. 4b,c) reduces the temperature rise as compared to the unmixed larger nanoparticles of the corresponding size. Further, comparison of the experimentally measured temperatures to the melting point of Ag nanoparticles (Fig. 4d, after Alarifi *et al.*^28^) shows that nanoparticle melting does not occur during the photonic sintering experiments performed here. These observations raise the following questions.

The conventional hypothesis in literature^16^,^17^,^18^ is that melting is the densification mechanism in photonic sintering, and faster densification of smaller nanoparticles is primarily due to the size-dependent depression in melting points. As shown by Fig. 4, and by the densities of the sintered material in Fig. 3, smaller nanoparticles can achieve higher densities despite the absence of melting. The alternate to nanoparticle melting is interparticle neck growth. Greater surface diffusion due to larger surface area per unit volume of smaller nanoparticles should accelerate densification for smaller nanoparticles. However, photonic heating in smaller nanoparticles can be orders of magnitude lesser than in larger nanoparticles, which should reduce the temperature rise and densification of smaller nanoparticles. The key questions is, can neck growth based densification capture the experimentally observed effects of nanoparticle size on temperature and density?

Furthermore, it is known that neck growth affects interparticle geometry and that nanoparticle shape affects photonic heating^22^,^23^,^24^,^25^,^26^,^27^. Thus, the second question is, if densification occurs by neck growth then is there a relationship between the evolution of neck growth and photonic heating? Within our knowledge, there is little work that investigates the existence or absence of coupling between neck growth and photonic heating in nanoparticle ensembles. Thirdly, in photonic sintering of mixed nanoparticle inks, why is an increase in densification observed with an increase in the weight ratio of smaller to larger nanoparticles? The next section describes a computational model we have developed to answer these questions.

### Modeling

**Figure 5:**
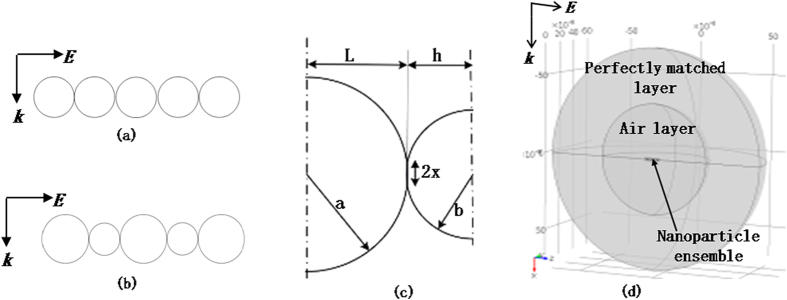
Schematic of model for (a) photonic sintering of equal sized nanoparticles (b) photonic sintering of unequal sized nanoparticles (c) geometric parameters describing interparticle necks (d) FEA of photonic heating.

A computational model was developed to couple photonic heating, the temperature rise in the nanoparticles and the kinetics of interparticle neck growth. The focus was on nanoparticle densification rather than evaporation of the solvent in the ink. To retain computational feasibility, while studying the above phenomenon on the nanoscale, the analysis domain consisted of five spherical three-dimensional in-plane nanoparticles of same or different sizes that touch each other (Fig. 5a,b).

To model interparticle neck growth the analytical McMee***k***ing-Kocks-Suo model^29^, which captures sintering and coarsening of rows of equal and unequal sized particles, was adapted to account for a continuous change in temperature without any external sintering pressure. At any instant of time the interface between two touching nanoparticles was described by the geometric parameters shown in Fig. 5c. Here *2x* is the neck size, *a* and *b* are the nanoparticle diameters, and *L* + *h* is the distance between the nanoparticle centers. When the two nanoparticles are of equal size then *a* = *b* and *L* = *h*. Additionally, a heat transfer model was incorporated to capture photonic heating induced temperature rise and radiative losses to the ambient. The wave optics module in COMSOL was used to perform frequency domain FEA simulations (Fig. 5d) to predict photonic heating power 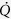 in the nanoparticle ensembles. The nanoparticle ensemble was embedded in a layer of air, surrounded by a perfectly matched layer. The symmetry of the problem was utilized to model one half of the system. The incident light was modeled in the frequency domain with polarization along direction *E* and direction of propagation along *k*. Based on the geometry of the fiber optic light guide, as supplied by the guide manufacturer, it was assumed that only 60% of the commanded optical power in experiments was incident on the deposited nanoparticles. Further details of both the analytical sintering model and the FEA model are described in the methods section.

Distinct FEA simulations, corresponding to different geometries of the interparticle necks in the ensemble, were performed to quantify 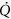 as an analytical function of the largest *x/b* ratio in the ensemble. To perform these FEA simulations, the stable intermediate neck geometries were obtained by using a constant dummy value of 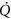 in the analytical sintering model and sampling the evolution of the geometric parameters. The FEA was performed for both unmixed (Fig. 5a) and mixed (Fig. 5b) nanoparticles. The analytical form of 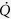 vs. *x/b*, for each nanoparticle ensemble, was then used by the analytical sintering model to predict the actual evolution of temperature and interparticle neck geometries during photonic sintering. The sintering portion of the analysis was performed until the *x/b* ratio at any interface became ≥0.9, indicating the forming of a pill-shaped structure with near complete densification at that interface. A conventional constant heating model was also developed, to examine the constant photonic heating assumption in literature, by forcing 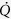 to be a constant value irrespective of neck geometry. This constant value corresponded to the 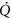 in the unsintered state, as predicted by the FEA.

### Model Predictions for Unmixed Nanoparticle Inks

**Figure 6:**
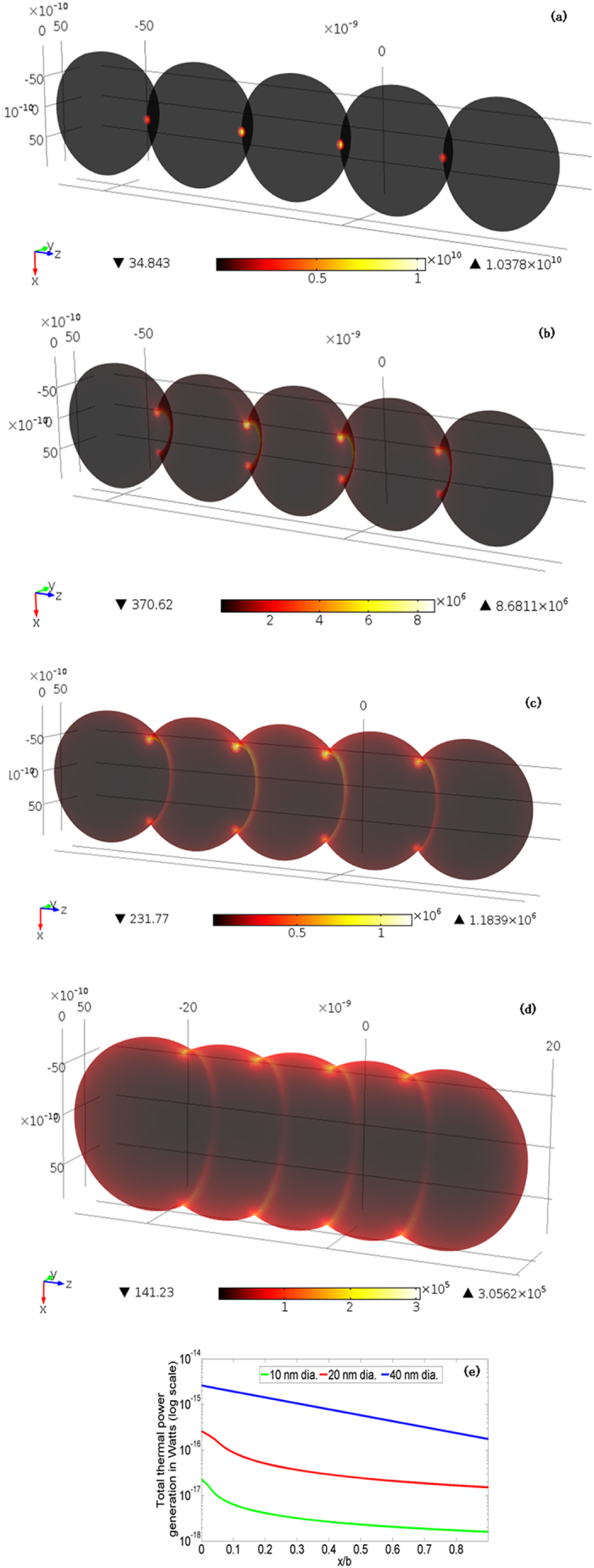
Thermal power density (in W/m^3^) for 20 nm nanoparticles and 1 V/m incident field at 400 nm wavelength for (a) *x/b* = 0 (b) *x/b* = 0.3 (c) *x/b* = 0.6 (d) *x/b* = 0.88; and (e) total thermal power as a function of *x/b* for 1 V/m incident electric field.

Figure 6a–d show representative contours of thermal power density as a function of *x/b*, for unmixed 20 nm nanoparticles with incident light corresponding to an electric field of 1 V/m and wavelength 400 nm. Contours for other unmixed nanoparticles are shown in Supplementary Figs S4 and S5 online. The thermal power density is highest at the interface between adjacent nanoparticles and its peak value reduces as the *x/b* increases. The predicted relationship between 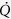 and *x/b* (Fig. 6e) shows that the FEA is able to capture the well known reduction in photonic heating with reducing nanoparticle size.

**Figure 7:**
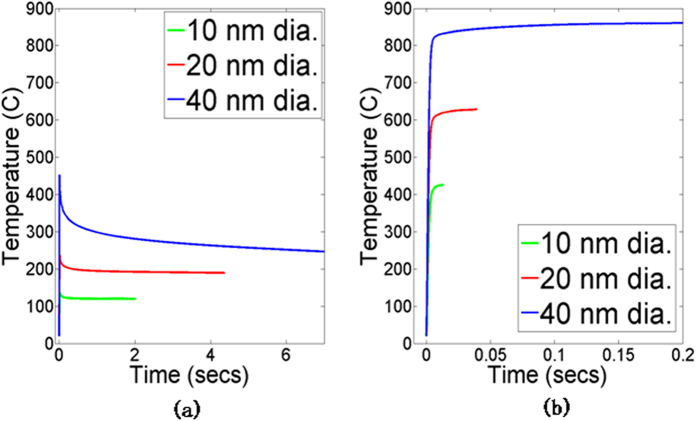
Predicted temperature evolution for unmixed inks (a) coupled model (b) constant heating model.

**Figure 8:**
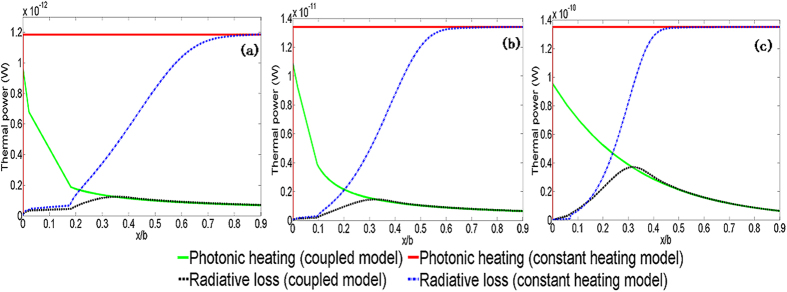
Comparisons of predicted photonic heating vs. radiative losses for (a) 10 nm unmixed inks (b) 20 nm unmixed inks (c) 40 nm unmixed inks.

**Figure 9:**
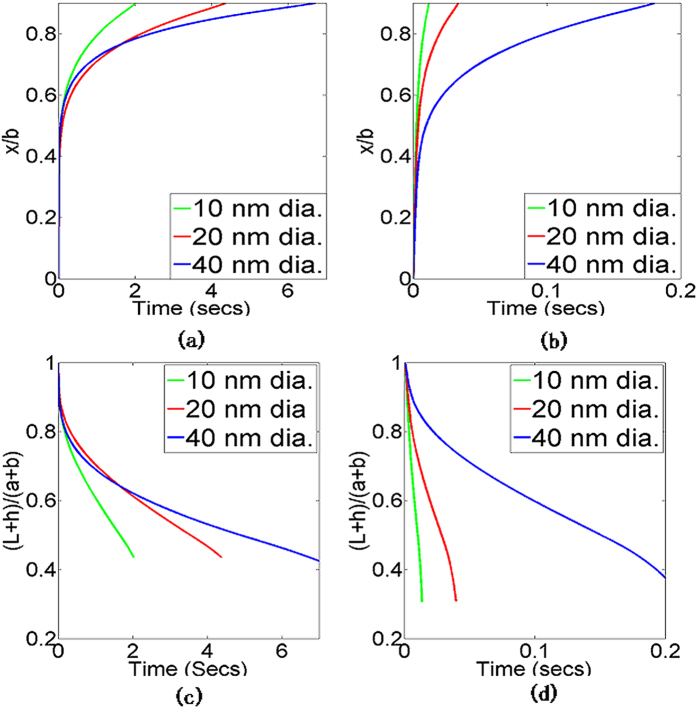
Predicted ratio x/b for unmixed inks from (a) coupled model (b) constant heating model; and predicted ratio (*L* + *h*)/(*a* + *b*) for unmixed inks from (c) coupled model (d) constant heating model.

At the same time, a new observation is that 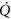 reduces by almost an order of magnitude as *x/b* increases. Since the progress of densification (with greater *x/b*) causes a reduction in subsequent photonic heating, therefore photonic sintering is a self-damping process. Figures 7, 8, 9 show model predictions corresponding to the total optical power incident on the nanoparticles during experiments. Figure 7 shows that the coupled model and the constant heating model capture the greater rise in temperature for larger nanoparticles, which is also seen in experiments. However, the experimentally observed trend (Fig. 4a) of a spike in temperature followed by stabilization to a steady state temperature as well as the peak temperature magnitudes, are better captured by the coupled model. For example, the constant heating model predicts that the 40 nm nanoparticles reach melting point, which is not seen experimentally and is also not predicted by the coupled model.

Comparison of the photonic heating and radiative losses (Fig. 8) shows that the self-damping nature of photonic sintering allows radiative losses to catch up with photonic heating at a much lower *x/b* (i.e., much earlier during densification) than if constant photonic heating was occurring. This explains the experimentally observed trend of a spike and then stabilization of temperature, as predicted by the coupled model. Figure 9 shows the predicted evolution of the ratios *x/b* and *(L* + *h*)/(*a* + *b*). Both coupled and constant heating models capture the faster densification of smaller nanoparticles. However, the constant heating model predicts orders of magnitude faster densification than the coupled model. Since the temperature trends, which drive densification, are significantly overestimated by the constant heating model it can be inferred that that the conventional constant heating model also overestimates the sintered material density.

At the same time, it should be noted that neither model captures the rate of temperature rise adequately. The peak temperature is reached orders of magnitude faster with both models, as compared to experimental observations. A possible reason is that only a small number of nanoparticles are being modeled and the smaller mass results in a much faster temperature rise. A mesoscale model that accounts for the coupling between neck growth and photonic heating may resolve this issue.

### Model Predictions for Mixed Nanoparticle Inks

**Figure 10:**
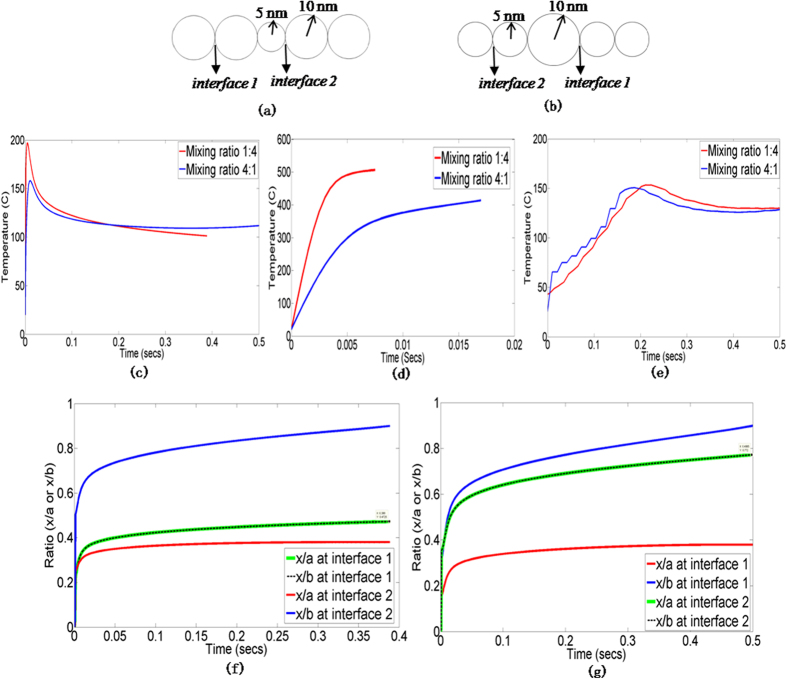
Domain geometry for (a) 1:4 case (b) 4:1 case; Temperature evolution from (c) coupled model (d) constant heating model (e) experiments; Ratios *x/a* and *x/b* at interfaces 1 and 2 for (f) 1:4 case (g) 4:1 case.

Figure 10a,b show the modeled geometry for the 1:4 and 4:1 weight ratios of 10 nm and 20 nm particles respectively, along with the interface nomenclature which will be used to understand model predictions.

The mixed nanoparticles in this model consist of 10 *nm* and 20 *nm* nanoparticles in ratios of 1:4 and 4:1 by number. While the resulting weight ratio of the mixed nanoparticles is not exactly the same as in experiments, these cases still let us examine the effect of increasing the weight ratio of smaller to larger nanoparticles. Mixing of the 10 nm and 20 nm nanoparticles in a ratio 4:1 by number has a weight ratio of 1/2 whereas mixing in a ratio 1:4 has a weight ratio of 1/32.

Representative thermal power density contours for mixed nanoparticles are shown in Supplementary Figs S6 and S7 online. Figure 10c–e compare the experimentally measured temperature evolution to predictions from the coupled model and the constant heating model, for the above mixed nanoparticle cases. As seen for unmixed nanoparticles, the experimentally observed trend of a peak and then stabilization in temperature as well as the peak temperature magnitudes (Fig. 10e), are captured much better by the coupled model (Fig. 10c) than by the constant heating model (Fig. 10d). Figure 10f,g show the evolution of *x/a* and *x/b* at interfaces 1 and 2. For each mixing ratio the *x/b* ≥ 0.9 condition is first reached at the interface between the smaller and larger nanoparticles, i.e., at interface 2 for the 1:4 case and interface 1 for the 4:1 case. This faster neck growth at the interface between dissimilarly sized particles has also been observed in earlier works on sintering^29^. For the 1:4 case (Fig. 10f) when *x/b* = 0.9 at interface 2 then *x/b* (=*x/a*) is 0.47 at interface 1. For the 4:1 case (Fig. 10g) when *x/b* = 0.9 at interface 1 then *x/b* (=*x/a*) is about 0.77 at interface 2. Thus, the ratio of *x/b* at the interface between similarly sized nanoparticles, when *x/b* = 0.9 is reached for the interface between dissimilar nanoparticles, is 1.6 times greater for the 4:1 case than for the 1:4 case. Thus, a larger weight ratio of smaller to larger nanoparticles results in the larger nanoparticles being embedded in a matrix of highly densified smaller nanoparticles and densification is faster for the 4:1 case.

## Discussion

In this work, we have experimentally characterized the densification and temperature evolution in photonic sintering of Ag nanoparticles, as a function of nanoparticle size. We have shown that smaller nanoparticles undergo faster densification despite lower temperatures during sintering, as compared to larger nanoparticles, and can do so without nanoparticle melting. The trend of a spike and then stabilization in temperature that is experimentally observed here is not seen in current literature on photonic sintering. This is because the typical duration of the optical pulse on-time in literature is about 10–15 ms, which is much smaller than the time taken to reach the maximum temperature in our experiments, i.e., around 500 ms. At the same time, the peak power density required for sintering in our experiments is no more than a few 100 W/cm^2^ whereas the corresponding value in literature is typically 1000 W/cm^2^ or greater. The impact of this observation is that lower optical power can be used to achieve high densification, rather than using excessive optical energy to ensure nanoparticle melting and thermally degrading the substrate in the process.

The coupled model developed to investigate the densification mechanism shows that photonic sintering is self-damping in nature, i.e., as neck growth and densification progress the amount of photonic heating reduces. By accounting for this self-damping effect this coupled model captures experimentally observed trends in temperature evolution much better than the conventional constant heating model, which overestimates both the temperature rise and densification during photonic sintering. Thus, the use of conventional models to control the power and on-time of the xenon lamp in practice can cause two issues. First, it can result in the use of expensive high-temperature substrates to prevent the higher temperatures predicted by the conventional model from damaging the substrate. Secondly, the overprediction of sintered density by conventional models can lead to excessive porosity and poor functionality of the sintered nanoparticles. We have also experimentally shown that for mixed nanoparticle inks densification is faster for higher weight ratio of smaller to larger nanoparticles. This is because a lower weight ratio results in fast coalescence of the smaller nanoparticles into the larger ones, but the larger nanoparticles are unable to coalesce with each other fast enough. To summarize, this paper uncovers a coupling between interparticle neck growth and photonic heating that should be included into mesoscale models of photonic sintering to prevent oversimplification of the process physics. Additional factors that may influence the process physics include the thermal properties of the substrate, thermal transfer into the bulk of the deposited nanoparticles and the effects of nanoparticle packing. Investigations into these areas are currently being conducted by the authors.

## Methods

### Photonic Sintering Experiments

A PerkinElmer XL300 continuous xenon lamp source was used as the light source for photonic sintering experiments. The distance between the outlet of the light guide and the substrate was fixed such that the light spot diameter on the substrate was 4 mm. One μL of each ink was deposited onto strips of mirror finish 304 stainless steel using a micropipette, and was exposed to a computer controlled commanded optical power of 300 W for a lamp on-time of 5 seconds. The nanoparticles were bought from Sigma Aldrich, suspended in tetradecane solvent, and ultrasonicated for two hours before sintering experiments were performed. A MicroEpsilon thermo-imager TIM 200 thermal camera was used to record temperature evolution during the sintering experiments. This camera records at a frequency of 128 Hz, with a spatial resolution of 96 pixels per inch at the substrate, and measures temperatures of upto 1500 °C with an accuracy of ±2 °C. The emissivity of the nanoparticles and the substrate were measured by raising the temperature of the substrate or nanomaterial to a constant 100 °C on a hot plate, measuring the steady state temperature using a thermocouple, and setting the emissivity so that the steady-state temperature readings from the thermocouple and the camera matched. The cross sectional SEM images in Fig. 3b,i were obtained after cutting the sintered Ag using FIB.

### Sintering model

The geometric parameters of the neck during photonic sintering are found by using Rayleigh-Ritz minimization of the functional in equation (1) where, *G*_*s*_ is the rate of change of the free energy of the system and *R*_*s*_ is one half the rate of energy dissipation in the system. The above extremization is performed under the constraints of volume conservation (equation 2) and geometric relationships (equation 3), after Parhami *et al.*^29^, and yields a system of linear equations (equation 4) in *a* and *b*. In equation (4), *d*_*b*_and *d*_*s*_are diffusion parameters for grain boundary and surface diffusion respectively, and are given by *d*_*b*_ = *δ*_*b*_*D*_*b*_*Ω/kT* and *d*_*s *_= *δ*_*s*_*D*_*s*_*Ω/kT*. Further, *δ*_*b*_ and *δ*_*s*_ are the thickness of the region with enhanced grain boundary and surface diffusion respectively, *D*_*b*_ and *D*_*s*_ are the grain boundary and surface atom diffusivities respectively, *Ω* is the atomic volume, *k* is the Boltzmann constant, and *T* is the temperature in Kelvin.


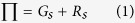


where, *a*_0_ and *b*_0_ are initial diameters of larger and smaller nanoparticles respectively


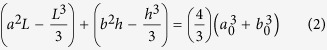



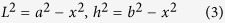


Both surface and grain boundary diffusion are considered here since these mechanisms dominate the sintering of nanoparticles^19^. We further assume that *δ*_*b*_ = *δ*_*s*_ and *D*_*b*_ = *D*_*s*_ = *D*_*0*_e^−*E/kT*^, where *E* is the activation energy and *D*_*0*_is a pre-exponential factor. The *γ*_*b*_and *γ*_*s*_ terms in equation (4) are grain boundary and surface energies per unit area respectively. The terms *g*_*a*_ = *ln*[*a/x*(*a* + √*1* − *x*^*2*^*/a*^*2*^)]−*L/a* and *g*_*b*_ = *ln*[*b/x*(*b* + √*1* − *x*^*2*^*/b*^*2*^)]−*h/b* are simplified geometric parameters that are used for ease of representation.


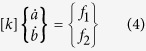


where *k* is a symmetric matrix with


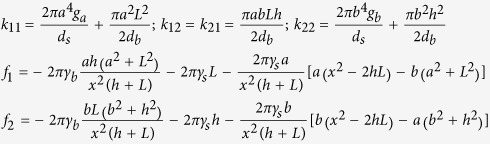



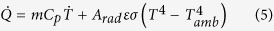



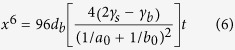


Initially, when the value of *x* is very small, the above solution procedure becomes unstable. To avoid this issue, we assume that ∂*a*/∂*t* = *∂b/∂t* = 0 till *x/b* > 0.01. This reduces the above formulation to a generalization of Coble’s law^29^, shown in equation (6), that governs the evolution of *x* with time *t* till *x/b* > 0.01. The temperature *T* is obtained via the heat transfer equation (equation 5) where 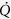 is the photonic heat generated in the nanoparticle ensemble, *m* is the total nanoparticle ensemble mass, *C*_*p*_ is the specific heat capacity of the nanoparticle material, *ε* is the emissivity of the nanoparticle material, *σ* is the Stefan-Boltzmann constant, *A*_*rad*_ is the surface area of the nanoparticle ensemble exposed to the ambient, and *T*_*amb*_ is the ambient temperature. *A*_*rad*_ is calculated from the geometric parameters and the initial nanoparticle radii *a*_*0*_ and *b*_*0*_, as shown in equation (7) for unmixed nanoparticles and in equation (8) for mixed nanoparticles.


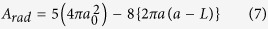



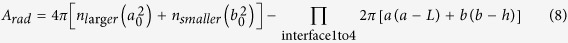


where, *n*_*l* arg*er*_ = number of larger NPs; *n*_*l smaller*_ = number of smaller NPs

Note that the duration of the transient conduction regime *τ* in a system is proportional to the characteristic length of the system^30^. For example, when *a* = *b* = 40 *nm* then *τ* = 26 *ns* for our system. Since the typical value of *τ* is much smaller than the duration of sintering, the temperature within the modeled nanoparticle ensemble can be assumed to be uniform, thus allowing transient heat conduction to be neglected in equation (5). The analytical neck growth model was implemented in MATLAB, using the material parameters shown in Table 1 below.

### Finite Element Model of Photonic Heating

The COMSOL FEA model (Fig. 5d) had a perfect magnetic conductor condition on the symmetrical planar faces of the model. The spherical layer of air and perfectly matched layer were of radii *λ/2* and *λ* respectively, where *λ* is the incident light wavelength. The electric field magnitude of the incident light was 1 *V/m*. Mesh convergence simulations were performed to eliminate spurious electric field reflections. The final mesh size used had a largest element size of 0.5 *nm* for the nanoparticles and of *λ*/15 for the air and perfectly matched layers. The wavelength dependent dielectric constants of silver were obtained from the Drude-Lorentz model^31^. The nanoparticle diameters in the model were the same as the nominal nanoparticle diameters used in experiments, i.e., 10 nm, 20 nm and 40 nm. The parametric analysis technique in COMSOL was used to vary *λ* from 400 *nm* to 700 *nm* for each FEA. This range was used since most of the optical power from the xenon lamp is concentrated within this wavelength range, as per the lamp manufacturer (see Supplementary Fig. S8 online). At each wavelength, the predicted thermal power density was integrated over the volume of the ensemble to obtain the thermal power generated in the ensemble. Thus analytical forms of thermal power as a function of wavelength *λ*, i.e., 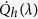, were obtained for each set of geometric neck parameters. Representative curves of 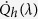, for unmixed nanoparticles, are shown in Supplementary Fig. S9 online. The total thermal power generated for each set of geometric parameters, i.e., 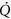, was computed using equation (9). The 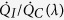 term was obtained from the wavelength dependent relationship between the incident power 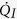 and the commanded power 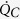, as supplied by the xenon lamp manufacturer (see Supplementary Fig. S9 online). The *W* term is the incident electric field (in V/m) corresponding to the commanded lamp power. Curve fitting was used to obtain 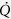 as an analytical function of the largest *x/b* in the ensemble. This analytical function was used to couple the photonic heating model with the analytical sintering model.


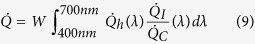


### Coupling between sintering model and photonic heating model

The evolution of the geometric parameters *a, b, x, L, h* was obtained via a time marching solution, as follows. At the beginning of a time increment, 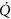 was obtained from the largest *x/b* in the ensemble by using the analytical function of 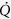 vs. *x/b* from the photonic heating model. Along with *A*_*rad*_, computed at the beginning of the time step from equation (7 or 8), equation (5) was used to compute the temperature rise in the ensemble via Forward Euler integration. If *x/b* < 0.01 then equation (8) was used to update *x*, and equations (2 and 3) were used to update *L* and *h*. If *x/b* > 0.01 then equation (4) was solved to find ∂a/∂t and ∂b/∂t, and the *a* and *b* were updated using Forward Euler integration. Equations (2 and 3) were then solved using a nonlinear equation solver to update *x, L* and *h*. In the next time step, the updated geometric parameters were again used to obtain 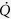 at the beginning of the next time increment.

## Additional Information

**How to cite this article**: MacNeill, W. *et al.* On the self-damping nature of densification in photonic sintering of nanoparticles. *Sci. Rep.* **5**, 14845; doi: 10.1038/srep14845 (2015).

## Supplementary Material

Supplementary Information

## Figures and Tables

**Table 1:** Model parameters used.

Parameter	Value
γ_b_	7.12 J/m^2^ 32
γ_s_	1.31 J/m^2^ 32
*δ*_*b*_ = *δ*_*s*_	0.5 nm^33^
Ω	10.27 cm^3^/mo^34^
*D*_*0*_	0.724 m^2^/s^33^
*E*_*a*_	45,500 J/mo^33^
*ε*	0.07
