# Supplementary material for: On the self-damping nature of densification in photonic sintering of nanoparticles
Source: Sci Rep. 2015 Oct 7;5:14845. doi: 10.1038/srep14845 (PMC4595846; doi:10.1038/srep14845)
Supplement: Supplementary Information [file srep14845-s1.pdf]

On the self-damping nature of densification in photonic sintering of nanoparticles

William Macneill, Chang-Ho Choi, Chih-Hung Chang and Rajiv Malhotra

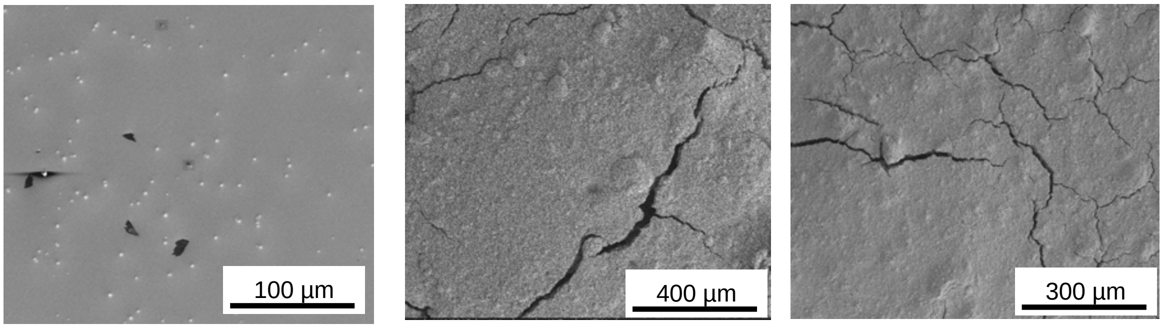

(a) (b) (c)

Supplementary Figure S1: SEM images of sintered (a) 10 nm (b) 20 nm (c) 40 nm inks.

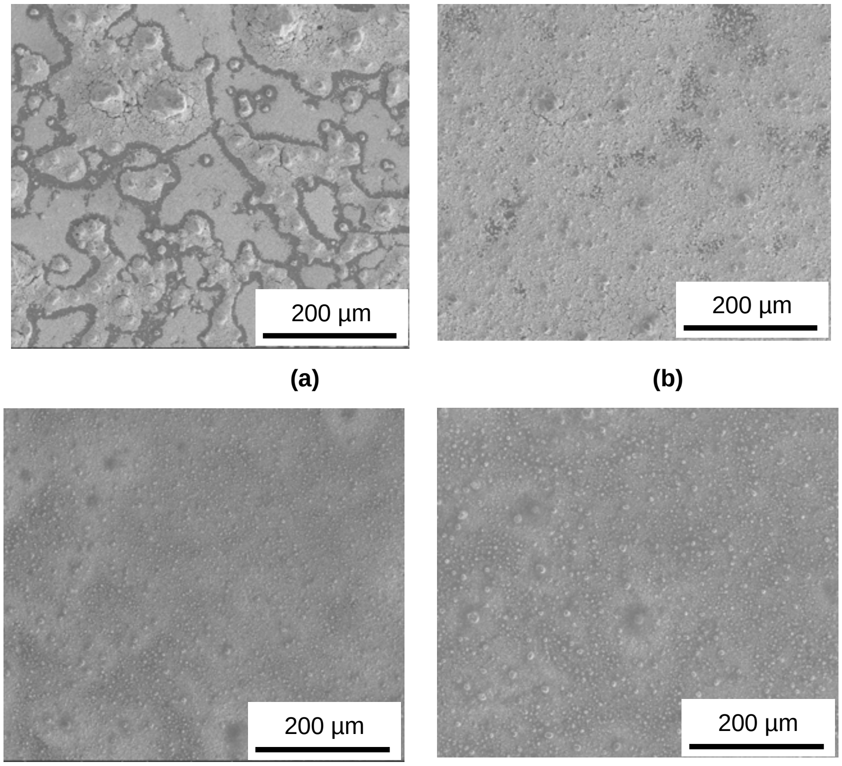

(a) (b) (c) (d)

Supplementary Figure S2: SEM images of sintered inks with 10 nm and 20 nm nanoparticles mixed in weight ratio (a) 1:4 (b) 2:3 (c) 3:2 (d) 4:1.

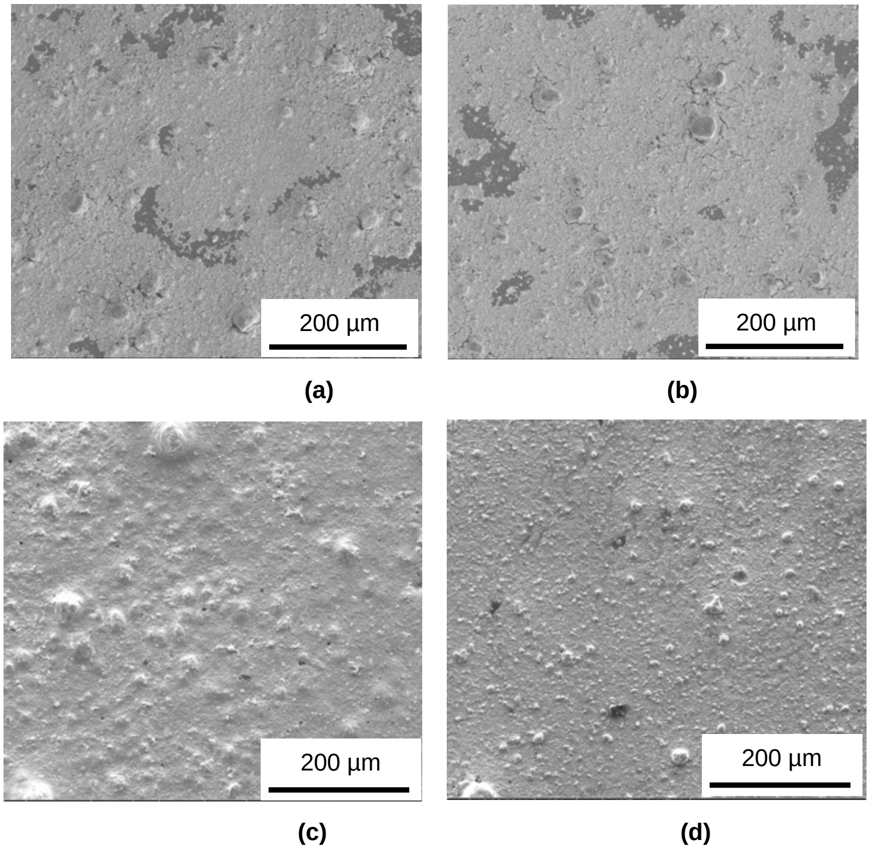

Supplementary Figure S3: 10 nm and 40 nm nanoparticles mixed in weight ratio (a) 1:4 (a) 1:4 (b) 2:3 (c) 3:2 (d) 4:1.

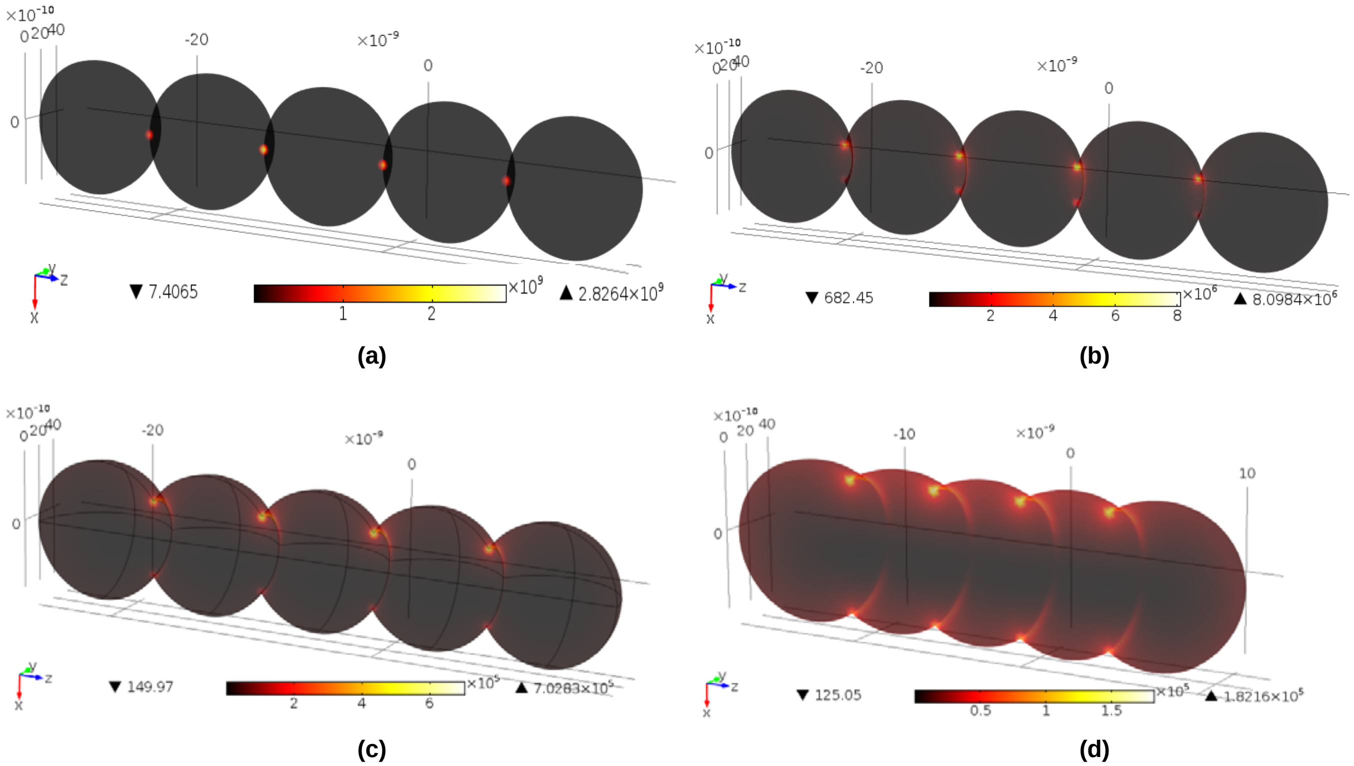

Supplementary Figure S4: Thermal power density (in W/m^3) for 10 nm nanoparticles and 1V/m incident field at 400 nm wavelength for (a) x/b = 0 (b) x/b = 0.26 (c) x/b = 0.63 (d) x/b = 0.85.

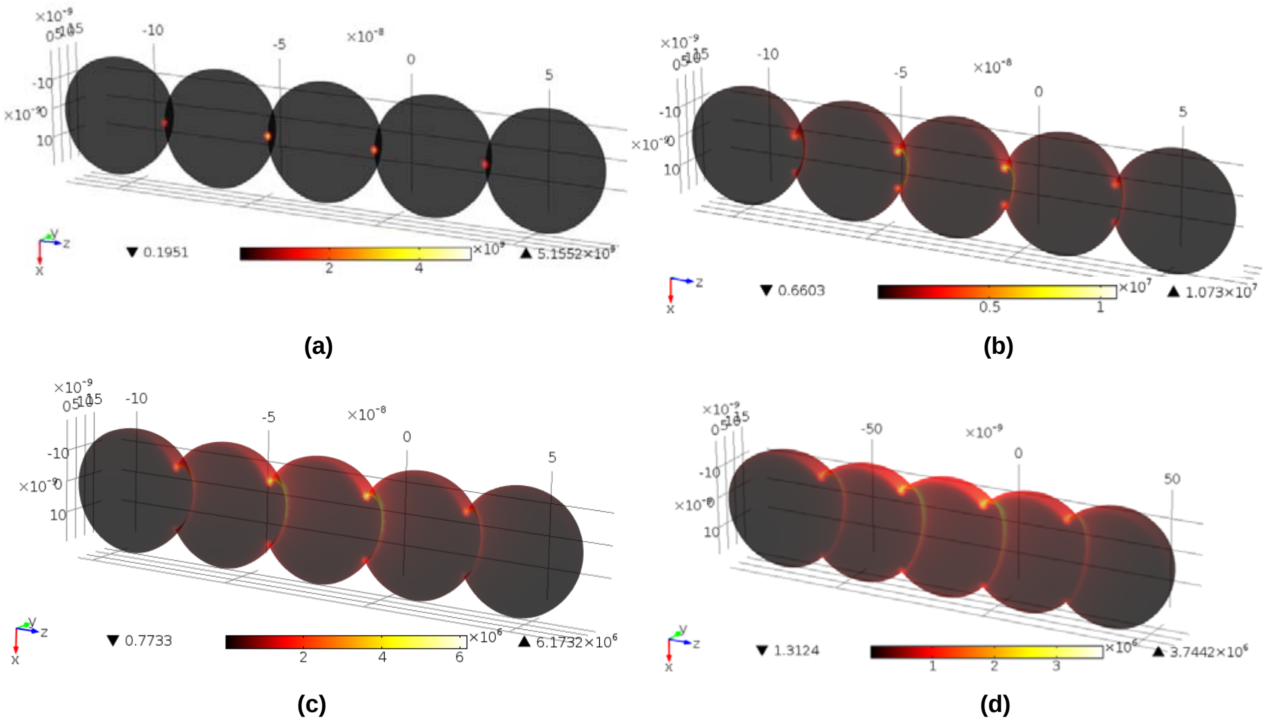

Supplementary Figure S5: Thermal power density (in W/m^3) for 40 nm nanoparticles and 1V/m incident field at 400 nm wavelength for (a) x/b = 0 (b) x/b = 0.3 (c) x/b = 0.6 (d) x/b = 0.85.

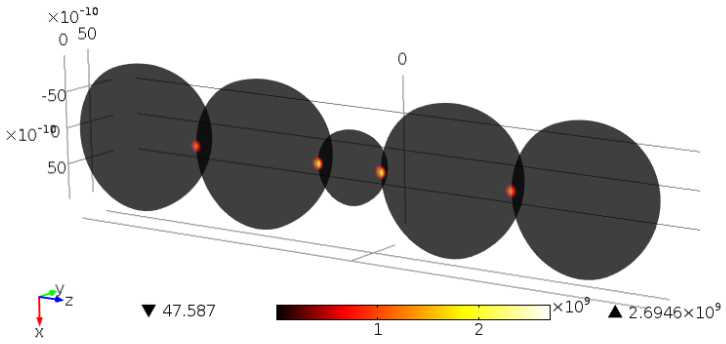

(a)

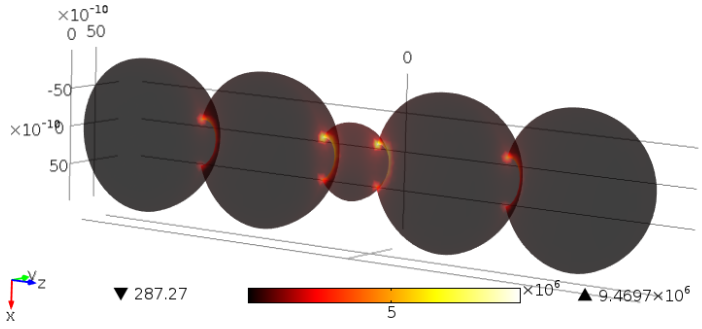

(b)

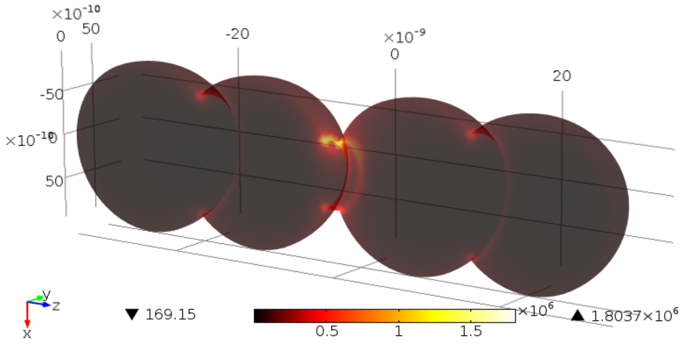

(c)

Supplementary Figure S6: Thermal power density (in W/m^3) for at 1V/m incident field and 400 nm wavelength for 10 nm and 20 nm nanoparticles mixed in ratio by number of 1:4 at (a) largest x/b = 0 (b) largest x/b = 0.54 (c) largest x/b = 0.90.

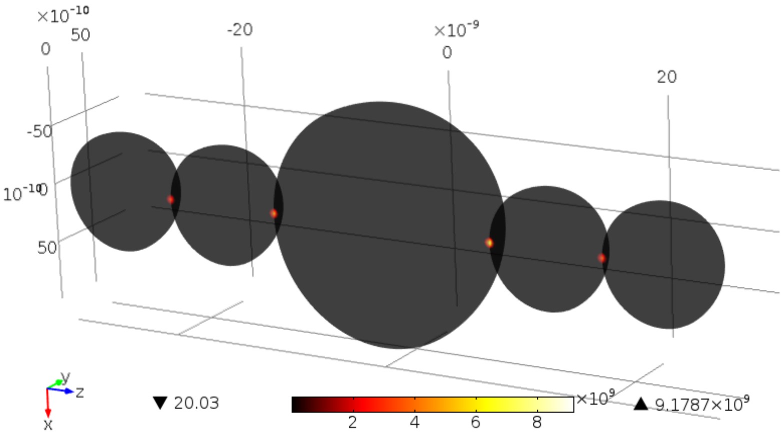

(a)

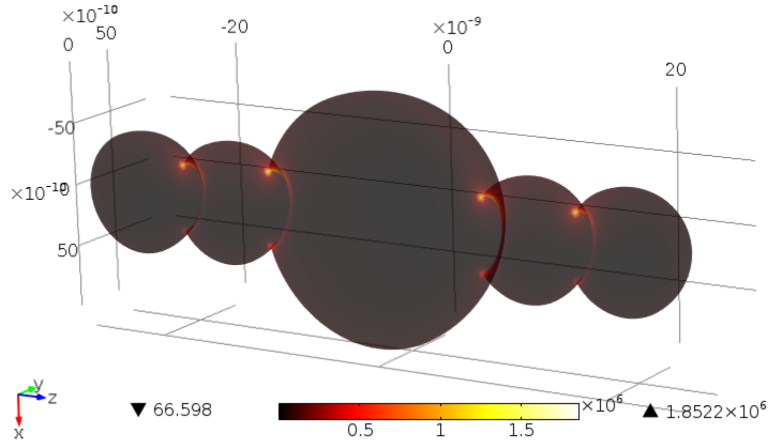

(b)

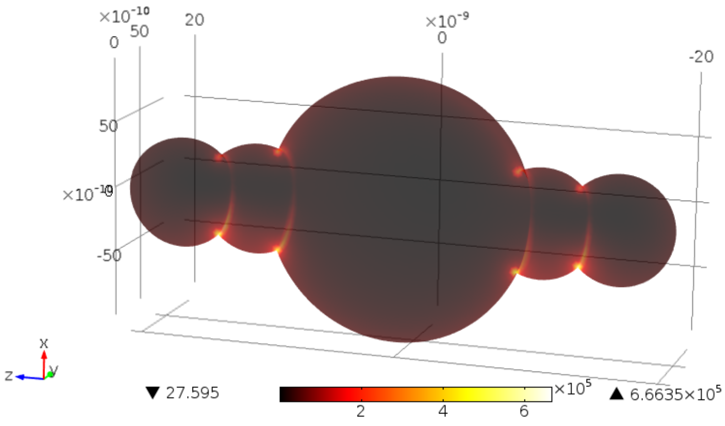

(c)

Supplementary Figure S7: Thermal power density (in W/m^3) for at 1V/m incident field and 400 nm wavelength for 10 nm and 20 nm nanoparticles mixed in ratio by number of 4:1 at (a) largest *x/b* = 0 (b) largest *x/b* = 0.6 (c) largest *x/b* = 0.86.

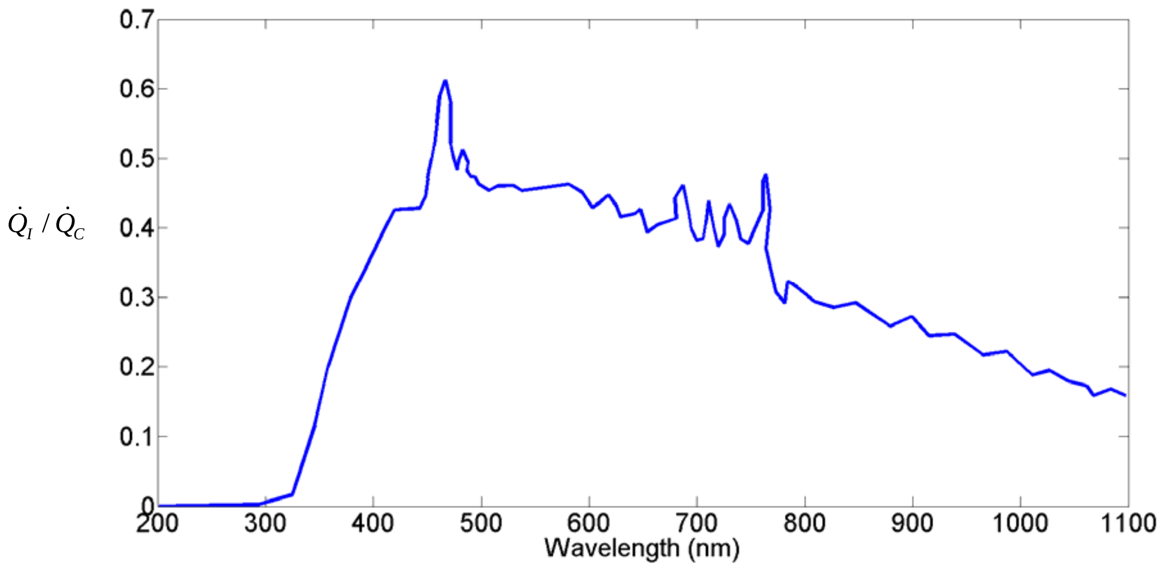

Supplementary Figure S8: Dependence of the ratio of incident xenon lamp power to commanded lamp power, i.e., $\dot{Q}_I / \dot{Q}_C(\lambda)$, as a function of the optical wavelength.

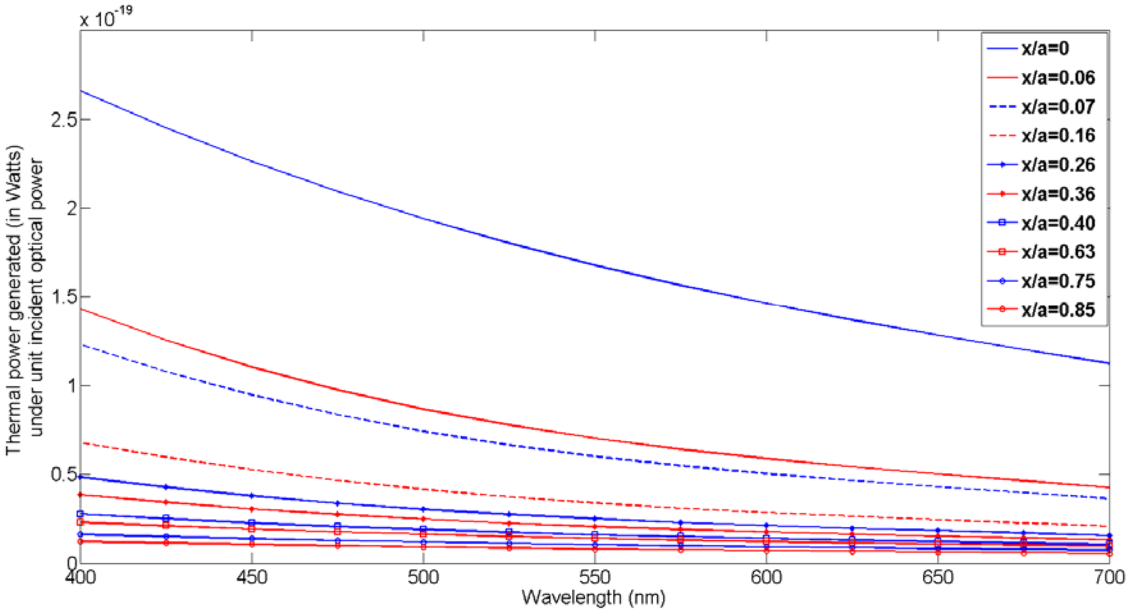
x 10^-19
Thermal power generated (in Watts) under unit incident optical power
Wavelength (nm)
x/a=0
x/a=0.06
x/a=0.07
x/a=0.16
x/a=0.26
x/a=0.36
x/a=0.40
x/a=0.63
x/a=0.75
x/a=0.85

(a)

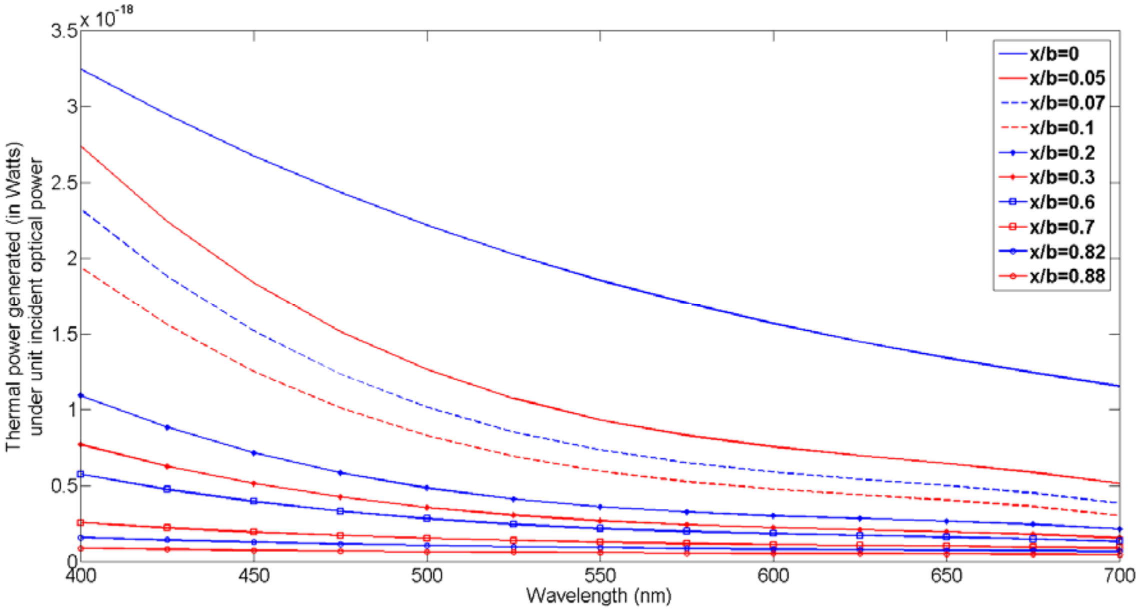
x 10^-18
Thermal power generated (in Watts) under unit incident optical power
Wavelength (nm)
x/b=0
x/b=0.05
x/b=0.07
x/b=0.1
x/b=0.2
x/b=0.3
x/b=0.6
x/b=0.7
x/b=0.82
x/b=0.88

(b)

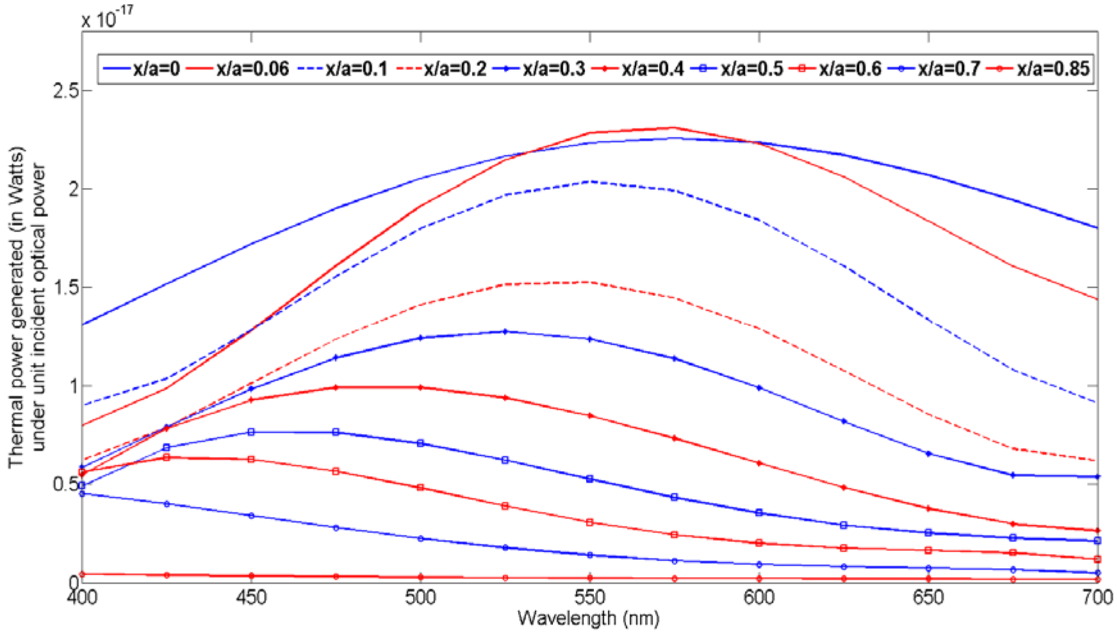

(c)

Supplementary Figure S9: Thermal power generated per unit incident optical power, i.e., $\dot{Q}_h(\lambda)$, for unmixed nanoparticles of diameters (a) 10 nm (b) 20 nm (c) 40 nm.
